# Infectious complications of extra-peritoneal pelvic packing in emergency room

**DOI:** 10.1007/s13304-020-00856-w

**Published:** 2020-08-10

**Authors:** E. Reitano, S. Granieri, S. Frassini, F. Sammartano, S. Cimbanassi, O. Chiara

**Affiliations:** 1 General Surgery and Trauma Team, ASST Niguarda, University of Milano, Piazza Ospedale Maggiore 3, 20162 Milano, Milan Italy; 2 General Surgery and Trauma Team, ASST Niguarda, Piazza Ospedale Maggiore 3, 20162 Milano, Milan Italy

**Keywords:** Emergency medicine, Extra-peritoneal pelvic packing, Hemodynamic instability, Pelvic fracture; emergency room

## Abstract

**Purpose:**

The Extra-Peritoneal Pelvic Packing (EPP) is a procedure used in emergency conditions to control pelvic hemorrhage. This procedure can be performed in Emergency Room (ER) if the patient is too unstable to be transported into the operating room (OR), with a possible increased risk of infections linked to a less sterile environment.

**Methods:**

All patients who underwent EPP from 2009 to 2018 were selected from the trauma registry. The patients were divided into two groups according to where EPP was performed (ER or OR). A Propensity Score Matching was realized. EPP was removed in all patients in the OR after obtaining hemodynamic stabilization within 24–48 h and surgical pads were sent to the laboratory for microbiological analysis.

**Results:**

Eighty-four patients underwent EPP during the period of the study. After PSM, 26 couples of patients were selected. No differences were observed between the two groups in the development of pelvic infection. Patients managed in OR showed a higher rate of associated abdominal injuries (*p* = 0.027) and an increasing need for external fixation (*p* = 0,005) as well as an increased proportion of laparotomies (*p* = 0.023), orthopedic interventions (*p* = 0.005) and a higher systolic blood pressure on admission (*p* = 0.003).

**Conclusions:**

The EPP is a safe procedure, even when performed out of OR. The EPP in ER allows an earlier control of bleeding in patients in extremis. To minimize the risk of infection, EPP should be removed early, as soon as hemodynamics have been stabilized.

## Headings

1. Extra-peritoneal pelvic pacing (EPP) is a procedure to control pelvic hemorrhages in emergency conditions
2. The main complication related to pelvic packing is represented by local infection
3. EPP is a safe procedure even if performed in emergency room.

## Introduction

Exsanguinating hemorrhage is the leading cause of death in patients with pelvic fractures within the first 24 h due to pelvic fracture itself or to coexisting associated injuries [1]. Logothetopulos [2] in 1926 introduced the concept of pelvic packing to stop the bleeding and Extra-Peritoneal Pelvic Packing (EPP) gained popularity in the 1990s as a life-saving technique in exsanguinating pelvic hemorrhage. The EPP was modified in Germany in 1994 by Pohlemann [3] and adopted by several groups in Europe and USA, as a procedure which can be performed in Emergency Room (ER) in patients in extremis or in the operating room (OR), in more stable conditions [4]. An Italian Consensus Conference in Bergamo in 2013 reiterated that EPP plays an important role in the acute management of the unstable pelvic fractures with hemorrhagic shock [5]. Mechanical pelvic stabilization and EPP were recognized as the most effective methods to control venous bleeding from venous plexus and from the fractured bony surface [6]. EPP has been demonstrated to be effective also in 15% of patients with arterial bleeding [5] who frequently remain unstable even after a temporary stabilization of the pelvis [7]. These patients are possible candidates also for angioembolization (AE), but this is a time-consuming procedure that requires the immediate availability of the interventional radiology staff [5]. Another method for emergency control of severe abdominal–pelvic hemorrhage is the placement of Resuscitative Endovascular Balloon for Occlusion of the Aorta (REBOA) [8] via vascular femoral access. However, the vascular access in hypotensive patient may be difficult and clinical evidence on the efficacy of REBOA is still debated, with some studies showing an increase in mortality [9].

The main complication related to pelvic packing is represented by local infection with a range of 15–35%, which increases when packing is removed after 48 h [2]. Burlew et al. reported cases of pelvic space infections post EPP, mainly occurring in patients with open fractures or those with associated bladder or bowel injury [10].

**Fig. 1:**
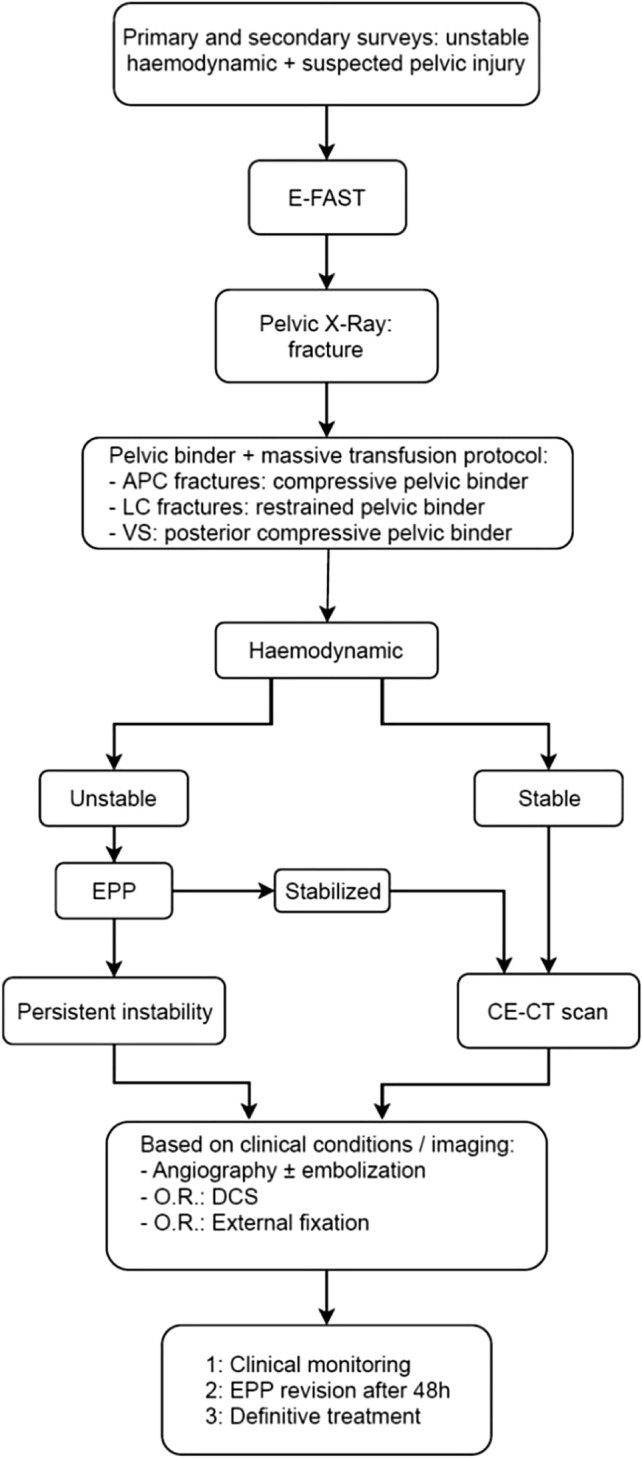
Treatment protocol for hemodynamically unstable patients with pelvic fracture. *APC* Anterior–posterior compression; *LC* lateral compression; *VS* vertical shear; *EPP* Extra-Peritoneal Pelvic Packing; *CE-CT* Contrast-enhancement computed tomography; *OR* Operating room

Since 2009, at our institution, the treatment protocol for patients with pelvic fractures and hemodynamic instability involves immediate EPP before laparotomy (if needed) followed by external fixation and angioembolization (AE) in case of persistent hypotension, or CT scan evidence of contrast spillage (Fig. 1).

The primary objective of our study was to evaluate if EPP in the emergency room (ER) is associated with a higher infection rate compared to the same procedure performed in the Operating Room (OR). Furthermore, we evaluated differences among patients who received EPP in ER and those packed in OR.

## Methods

All patients consecutively admitted to the Niguarda Trauma Center, a level 1 Trauma Center in Milan, Italy, from January 2009 to December 2018, with pelvic fracture and hemodynamic instability not responding to initial resuscitation, who underwent EPP, were selected from the trauma registry. Exclusion criteria were represented by head injury AIS ≥ 3 to avoid bias in the evaluation of mortality rate, previous organ transplantation, chemotherapy for neoplastic disease, chronic corticosteroid therapy and pelvic open fractures. All cases fulfilling these criteria were deemed eligible. Demographic data, vital signs in Emergency Department, Injury Severity Score (ISS), Abdominal Organ Injury Scale (OIS), number of packed red blood cells (PRBCs), rate of pelvic infections, death probability estimated using Trauma Injury Severity Score (TRISS score), surgical procedures other than EPP performed during the first 24 h (exploratory laparotomy, thoracotomy, orthopedic and vascular interventions, angioembolizations), diabetes, and survival outcome, were collected and retrospectively analyzed.

Hemodynamic instability was defined as persistent systolic blood pressure (SBP) of < 90 mmHg during initial resuscitation despite pelvic binder, pre-hospital fluids and transfusion of ≥ 2 units of PRBCs. High-energy pelvic fracture was defined as a fracture with horizontal (Tile B) or total (Tile C) mechanical instability, or fracture of the iliac wing (Tile A) in patients older than 75 [11]. EPP consisted in the positioning of 2–3 surgical pads on each side of the bladder, below the pelvic rim, as describe in a previous paper (six).

Packing was removed in all the patients in the OR after hemodynamic stabilization and surgical pads were sent to the laboratory for microbiological analysis. The removal of the pads always occurred within 24–48 h, according to our hospital protocol. Samples of the pads were squeezed, and the fluid was collected in an adequate transport terrain. Samples were timely sent to the laboratory for microbiological analysis.

Patients were divided into two groups according to where the EPP was performed (OR vs. ER). To estimate whether the EPP was related to a higher infectious risk, the development of pelvic infection of packing was defined as a microbiological contamination of the fluid collected from the pads, leukocytosis (> 15 × 10^3^/mL), fever (> 38 °C), elevated C-reactive protein (CRP) (> 10 mg/L) [12,13]. Microbiological contamination of the pads associated with at least one of the other aforementioned criteria was claimed for the diagnosis of pelvic infection. These criteria should be present at the time of pad removal, therefore within 48 h. None of the patients died within 5 days after removal of pads. Therefore, the development of infection has been assessed in all patients.

Data were recorded in a computerized spreadsheet (Microsoft Excel 2016; Microsoft Corporation, Redmond; WA) and analyzed with statistical software (IBM Corp. Released 2017. IBM SPSS Statistics for Windows, Version 25.0. Armonk, NY: IBM Corp.).

Propensity Score Matching (PSM) was then performed to adjust for differences in the baseline characteristics in the two groups. A one-to-one, nearest neighbor, logistic regression matching model was built setting the maximum tolerated difference between matched subjects (caliper) at 0.2 standard deviation (SD). ISS, RTS, and Tile pattern of fracture were selected as confounders. Graphical and mathematical (standardized differences, Iacus, King and Porro L1 test) balance diagnostics were evaluated after matching for an accurate assessment of the goodness of our model.

Results were reported as means/standard deviation or absolute values/percentages. Differences in proportions were compared using Chi-Square or Fisher’s test; whereas for continuous variables, Mann–Whitney test was performed. A *p* value < 0,05 was considered statistically significant.

## Results

Six hundred and seventy-five patients were admitted in our trauma center for pelvic fracture and 84 (12.44%) underwent EPP. The procedure was performed in ER in 42 cases and in OR in the other 42.

Main characteristics of the population of the study are reported in (Table 1). The mean age was 52.24 ± 19.84 years and most patients were men (59.5%). The most common mechanism of trauma was fall from height (38/84).

**Table 1:** Clinical characteristics of the sample and comparison among groups

Variables	EPP in ER (42)	EPP in OR (42)	*p* value
Gender (F/M) [*n* (%)]	19 (45.2)/23 (54.8)	15 (35.7)/27 (64.3)	0.505
Age (year) [mean (SD)]	52.29 ± 19.66	52.19 ± 20.26	0.851
Mechanism of trauma [*n* (%)]			0.056
Fall from height	24 (57.1)	14 (33.3)	
Pedestrian	9 (21.4)	9 (21.4)	
Motorcycle crash	3 (7.1)	13 (31)	
Motor vehicle crash	3 (7.1)	4 (9.5)	
Cycling crash	3 (7.1)	2 (4.8)	
Associated abdominal injuries [*n* (%)]	28 (66.7)	36 (85.7)	0.071
Systolic blood pressure (SBP) in ED [mean (SD)]	51.78 (28.44)	80.14 (20.24)	< 0.001*
PRBCs in 24 h [mean (SD)]	7.38 (4.85)	11.4 (11.49)	0.081
Pattern of pelvic fracture [*n* (%)]			0.329
Tile A	2 (4,8)	0	
Tile B	8 (19)	10 (23.8)	
Tile C	32(76.2)	32 (76.2)	
Surgical procedure other than EPP [*n* (%)]			0.861
Laparotomy	12 (28.6)	21 (50)	0.073
Thoracotomy	3 (7.1)	3(7.1)	1
Orthopedic surgery	27 (64.3)	40 (95.2)	0.001*
Vascular surgery	1 (2.4)	3(7.1)	0.616
Angioembolizations	20 (47.6)	24 (57.1)	0.512
Diabetes [*n* (%)]	3 (7.3)	5 (11.9)	0.713
ISS [mean (SD)]	51.38 (15.2)	43.48 (9.8)	0.007*
RTS [mean (SD)]	4.49 (2.06)	6.02 (1.72)	< 0.001*
Probability of death (TRISS) [mean (SD)]	56.92 (35.05)	40.82 (30.66)	0.016*

*EPP* Extraperitoneal Pelvic Packing; *PRBCs* Packed Red Blood Cells; *TRISS* Trauma and Injury Severity Score; *SD* Standard deviation

*Significant value

Considering the whole sample, patients managed in ER showed a higher ISS (*p* = 0.006) and probability of death (0.028), a lower RTS (*p* < 0.001), SBP (*p* < 0.001) and proportion of orthopedic interventions (*p* = 0.001) compared to OR group. No differences among groups were noticed regarding the incidence of diabetes.

**Fig. 2:**
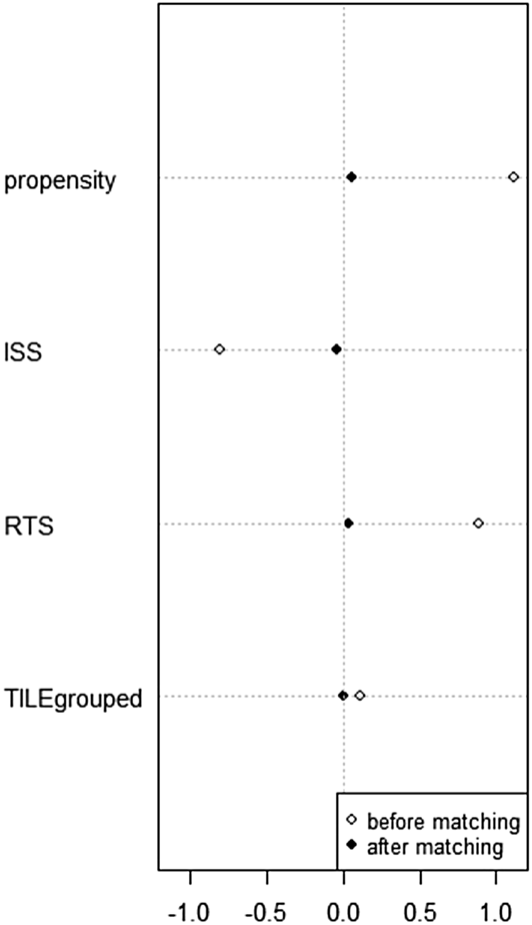
Dot plot of standardized differences. *ISS* Injury Severity Score; *RTS* Revised Trauma Score

Twenty-six couples of patients resulted eligible after Propensity Score Matching. Graphical assessment of balance before and after matching is displayed in (Fig. 2). For a more objective evaluation of balance diagnostic, we computed standardized differences of the selected confounders. For all covariates, we observed small effect size, defined by a *Cohen’s d* value below 0.2, after matching (Table 2). Furthermore, relative multivariate imbalance L1 test showed decreasing measurements (0.917 before matching; 0.73 after matching).

**Table 2:** PSM detailed balance

	Means treated		Means control		SD control		Std. Mean Diff	
	Before	After	Before	After	Before	After	Before	After
Propensity	0.604	0.520	0.396	0.509	0.226	0.189	1.117	0.058
ISS	43.476	46.231	51.381	46.615	15.203	14.911	− 0.806	− 0.039
RTS	6.027	5.449	4.491	5.382	2.059	1.721	0.890	0.039
TILE pattern of fracture	2.762	2.731	2.714	2.731	0.554	0.604	0.110	0.000

*ISS* Injury Severity Score; *RTS* Revised Trauma Score

Considering the whole sample, only 7 out of 83 patients (8.3%) developed pelvic infections. After PSM, two patients were excluded by the computation. All infections were sustained by gram-positive bacteria: Staphylococcus in four cases, Enterococcus in one case and Streptococcus in two cases. No differences among groups in terms of incidence of pelvic infection have been demonstrated (*p* = 1). Patients who underwent EPP in OR showed a higher incidence of associated abdominal injuries (*p* = 0.027). More in detail: six patients suffered from mesenteric injuries, six from hepatic injuries, four from splenic injuries, three from intestinal injuries, seven from bladder injuries, six from vascular injuries and three from adrenal gland injuries.

Similarly, patients treated in OR had a greater proportion of laparotomy and orthopedic interventions (*p* = 0,023 and *p* = 0,005 respectively), an increased need for external fixation (*p* = 0,005) and a higher SBP on admission. Further results are reported in (Table 3).

**Table 3:** Comparisons between EPP performed in ER and or after PSM

Variables	EPP in ER (26)	EPP in OR (26)	*P* value
Pelvic infections [*n* (%)]	2 (7.7)	3 (11.5)	1
Associated abdominal injuries [*n* (%)]	15 (57.7)	23 (88.5)	0.027*
Angioembolizations [*n* (%)]	13 (50)	14 (53.8)	1
External fixations [*n* (%)]	16 (61.5)	25 (96.2)	0.005*
Definitive fixations [*n* (%)]	8 (30.8)	12 (43.2)	0.393
Systolic blood pressure (SBP) in ED [mean (SD)]	56.27 (24.59)	79.7 (22.39)	0.003
PRBCs in 24 h [mean (SD)]	7.54 (5.23)	12.15 (11.71)	0.053
Surgical procedure other than EPP [*n* (%)]			0.861
Laparotomy	6 (23.1)	15 (57.7)	0.023*
Thoracotomy	2 (7.7)	2 (7.7)	1
Orthopedic surgery	16 (61.5)	25 (96.2)	0.005*
Vascular surgery	0 (0)	2 (7.7)	0.49
Angioembolizations	13 (50)	14 (53.8)	1
Probability of death (TRISS) [mean (SD)]	42.36 (30.46)	48.37 (33.11)	0.654
Length of hospitalization [mean (SD)]	38.46 (42.88)	51 (57.12)	0.352
Total deaths [*n* (%)]	12 (46.2)	11 (42.3)	1

*EPP* Extra-peritoneal Pelvic Packing; *PRBCs* Packed Red Blood Cells; *TRISS* Trauma and Injury Severity Score; *SD* Standard deviation

*Significant value

In all patients, the infections were managed with antibiotic therapy and no infection-related deaths were observed.

## Discussion

Extra-peritoneal pelvic packing was associated with a 50% increase in survival when compared to a population treated without this technique [14–16]. EPP is indicated as a life-saving procedure also in peripheral hospitals to stabilize the patient to allow the transfer to a higher-level center [14].

Performing EPP in an environment less sterile than the OR could theoretically lead to an increased rate of infections with a possible delay in subsequent orthopedic intervention for definitive stabilization. Available data about possible complications of pelvic packing are lacking in the literature. The study of Papakostidis et al. [16] estimated a high percentage of infections (35%) that was correlated with the presence of pads in the pelvis for few days (acting as foreign bodies) and with the impairment of immune defense mechanisms in critically ill patients [17]. Totterman et al. pointed out an infection rate of 33% in patients who received pelvic packing and described a case of severe sepsis in one patient in whom the pads remained in the pelvis for more than 3 days due to logistical problems in the removal [18]. In our patients, the removal of the pads after EPP was always performed in OR within 48 h and the pads were sent to the laboratory for microbiological analysis. When EPP was removed the surgeon explored the pelvis looking for residual bleeding, controlled with suture, topical agents or electrocautery and rarely repacking [6]. Burlew’s review demonstrated that repacking was associated with a higher percentage of infections and that risk factors for pelvic infections were open fractures, hollow viscous injuries and perineal wounds [10]. In our series, the presence of open fractures was one of the exclusion criteria and none of the patients who developed pelvic infection had concomitant hollow viscous injuries.

In the present study, the overall rate of pelvic infections after EPP was only 8.33% and no differences between ER-EPP and OR-EPP were noticed neither before nor after propensity score matching.

In our experience, the development of pelvic infections was not correlated with the environment where EPP was performed. Patients who underwent EPP in ER reported more severe injuries (as demonstrated by the higher ISS, probability of death and observed deaths and the lower RTS) compared to the OR-EPP group. On the other hand, this latter group was characterized by a higher percentage of associated abdominal injuries, need for laparotomy and orthopedic interventions. As part of our protocol, in case of an E-FAST positive for free abdominal fluid, the patient was transported to the operating room to treat both the abdominal and pelvic causes of hemodynamic instability by EPP first and then laparotomy. The necessity to perform other surgical procedures in OR represented one of the leading criteria in the selection of treatment strategy.

This study presents several limitations. Although our institution is a high-volume level 1 Trauma Center, pelvic bleeding with hemodynamic instability is a relatively rare condition, resulting in a limited sample size that unavoidably affected the power and goodness of our calculations. Moreover, our attempt to adjust for differences in the baseline characteristics using Propensity Score analysis resulted in a further reduction of the sample size.

Furthermore, we could not effectively assess the potential confounding effect of general risk factors of infection such as diabetes due to the extremely low incidence in our series.

On the other hand, to our knowledge, this is the first study that specifically investigates the problem of environment-related EPP infections.

In our hospital, the EPP was systematically performed in OR until 2013. After the implementation of the protocol it has become possible to perform EPP even in ER. The ER group was characterized by a lower systolic blood pressure, necessity of orthopedic interventions and external fixation as shown in (Table 3). The possibility to perform EPP in ER allowed a more rapid treatment of these critical patients without increasing the risk of infection.

## Conclusions

In conclusion, EPP is a safe, rapid and effective procedure that can be performed in ER. Performing EPP in ER could be useful in critically injured patients to obtain faster stabilization and to reduce the management time of life-threatening injuries. Data from studies with a larger cohort are mandatory for definitive conclusions about the balance of risks and benefits of EPP, compared to other strategies of pelvic hemorrhage control.

## Abbreviations

EPP: Extra-peritoneal Pelvic Packing
ER: Emergency Room
OR: Operating Room
ISS: Injury Severity Score
RTS: Revised Trauma Score
PD: Probability of death.
AE: Angioembolization
REBOA: Resuscitative Endovascular Balloon for Occlusion of the Aorta
CRP: C-reactive protein
OIS: Abdominal Organ Injury Scale
TRISS Score: Trauma Injury Severity Score
SBP: Systolic blood pressure
PRBCs: Packed red blood cells
